# That Woodbridge Treatment for Typhoid Fever

**Published:** 1898-08

**Authors:** 


### EDITORIAL DEPARTMENT.

F. E. DANIEL, M. D., Editor.
S. E. HUDSON, M. D., Managing Editor.
A. J. SMITH. M. D., Galveston, Associate Editor.

Published Monthly at Austin, Texas, by Drs. Daniel and Hudson. Subscription price $1.00 a year in advance.

Eastern Representatives: Messrs. Monihan & Fairchild, St. Paul Building, 220 Broadway, New York City.

Official organ of the West Texas Medical Association, the Houston District Medical Association, the Austin District Medical Society, the Brazos Valley Medeal Association, the Galveston County Medical Society, and several others.

### EDITORIAL.

THAT WOODBRIDGE TREATMENT FOR TYPHOID FEVER.—In our last issue I said in effect that a treatment that required a patient to be disturbed every fifteen minutes to take medicine; to be waked up to take medicine is a rank absurdity, and I say so yet; if there is any such treatment in vogue.

Every physician recognizes the value and importance of sleep in sickness, as well as in health. Young calls it "tired nature's sweet restorer." It is as essential to recuperation as is nutrition; indeed, one who does not sleep does not properly nourish. This is the only feature of the Woodbridge treatment that struck me as absurd; for the treatment—the disinfection of the alimentary tract, is based on rational principles, and is in accord with my views often expressed in these pages—especially of Dr. L. W. Cock's remedy for consumption. (See Red Back for April, 1897.) The desideratum in all diseases of bacterial infection is—to find a remedy which will destroy the germs or bacilli, without destroying also the patient, or harming him.

Parke, Davis & Co., the well known pharmacists who prepare the remedies from Dr. Woodbridge's formula--ready to the physicians' hand—have written to me calling attention to the fact that it is not a part of the Woodbridge treatment to administer the medicine except during waking hours. As the Irishman said who had whipped a Jew "for killing Christ," when told that it happened two thousand years ago—"its the first I've heard of it " so say I about the W. T. for T. F.; every publication I have seen on the subject stated that "No. 1"—was to be given every fifteen minutes.

The statement made by Messrs. Parke, Davis & Co., and the details of Dr. Woodbridge's plan which they send us, and which I have reproduced herewith, show that I was laboring under a misapprehension of fact, and hence take all the wind out of my sails. It is far from my wish or intention to be unjust or unfair, and I make this statement with pleasure. If the W. T. for T. F. is a good thing—and if it were not—experience would soon condemn it, "push it along." Hudson says it is a good thing, and he uses it. He says that until the treatment has been used, sleep is impossible, anyway; that after the disinfection of the bowels the patient sleeps sweetly, etc. I have never used it; don't practice in t. f.; don't have to, and I'm glad of it.

I think, as I have often said, that if the doctors would lay in a supply of pharmacal products, such as all reputable houses are now supplying—a list embracing every popular or tested and approved combination of remedies—in concentrated form, and would dispense them, thus doing away with the prescription druggist, the middle (and superfluous) man, it would be money in their pockets, and in the pockets of their patients; for when one of them gets well he has such a big drug bill to pay usually, that he *can't pay the doctor*, or *will* not, for spite, (and serves him right, too). "Its this regard that makes (homeopathy) calamity (well, they are synonymous) of so long life." (See Hamlet). The widow has no drug bill to pay—see?

